# Delayed headache after COVID-19 vaccination: a red flag for vaccine induced cerebral venous thrombosis

**DOI:** 10.1186/s10194-021-01324-5

**Published:** 2021-09-17

**Authors:** David García-Azorín, Thien Phu Do, Andreas R. Gantenbein, Jakob Møller Hansen, Marcio Nattan P. Souza, Mark Obermann, Heiko Pohl, Christoph J. Schankin, Henrik Winther Schytz, Alexandra Sinclair, Guus G. Schoonman, Espen Saxhaug Kristoffersen

**Affiliations:** 1 Headache Unit, Department of Neurology, Hospital Clínico Universitario de Valladolid, Valladolid, Spain; 2 The Danish Headache Center, Department of Neurology, Rigshospitalet-Glostrup, Faculty of Health Sciences, University of Copenhagen, Glostrup, Denmark; 3 Department of Neurology and Neurorehabilitation, ZURZACH Care, Bad Zurzach, Switzerland; 4 Department of Neurology, University Hospital Zurich, Zürich, Switzerland; 5 Danish Headache Center, Rigshospitalet-Glostrup, Faculty of Health Sciences, University of Copenhagen, Glostrup, Denmark; 6 Department of Neurology, Hospital das Clínicas, University of São Paulo, São Paulo, Brazil; 7 Department of Neurology, Hospital Weser-Egge, Höxter, Germany; 8 Department of Neurology, University of Duisburg-Essen, Essen, Germany; 9 Department of Neurology, University Hospital Zurich, Zurich, Switzerland; 10 Department of Neurology, Inselspital, Bern University Hospital, University of Bern, Bern, Switzerland; 11 Metabolic Neurology, Institute of Metabolism and Systems Research, College of Medical and Dental Sciences, University of Birmingham, Birmingham, B15 2TT UK; 12 Department of Neurology, University Hospitals Birmingham NHS Foundation Trust, Birmingham, B15 2TH UK; 13 Department of Neurology, Elisabeth-TweeSteden Hospital, Tilburg, The Netherlands; 14 Department of Neurology, Akershus University Hospital, Lørenskog, Norway; 15 Department of General Practice, HELSAM, University of Oslo, Oslo, Norway

**Keywords:** Headache disorders, Secondary; sinus thrombosis, Vaccine-induced immune thrombotic thrombocytopenia, Cerebrovascular disorders; Stroke

## Abstract

**Background:**

Headache is a frequent symptom following COVID-19 immunization with a typical onset within days post-vaccination. Cases of cerebral venous thrombosis (CVT) have been reported in adenovirus vector-based COVID-19 vaccine recipients.

**Findings:**

We reviewed all vaccine related CVT published cases by April 30, 2021. We assessed demographic, clinical variables and the interval between the vaccination and onset of headache. We assessed whether the presence of headache was associated with higher probability of death or intracranial hemorrhage.

We identified 77 cases of CVT after COVID-19 vaccination. Patients’ age was below 60 years in 74/77 (95.8%) cases and 61/68 (89.7%) were women. Headache was described in 38/77 (49.4%) cases, and in 35/38 (92.1%) was associated with other symptoms. Multiple organ thrombosis was reported in 19/77 (24.7%) cases, intracranial hemorrhage in 33/77 (42.9%) cases and 19/77 (24.7%) patients died. The median time between vaccination and CVT-related headache onset was 8 (interquartile range 7.0–9.7) days. The presence of headache was associated with a higher odd of intracranial hemorrhage (OR 7.4; 95% CI: 2.7–20.8, *p* < 0.001), but not with death (OR: 0.51, 95% CI: 0.18–1.47, *p* = 0.213).

**Conclusion:**

Delayed onset of headache following an adenovirus vector-based COVID-19 vaccine is associated with development of CVT. Patients with new-onset headache, 1 week after vaccination with an adenovirus vector-based vaccine, should receive a thorough clinical evaluation and CVT must be ruled out.

**Supplementary Information:**

The online version contains supplementary material available at 10.1186/s10194-021-01324-5.

## Introduction

Headache is within the most frequent adverse effects following coronavirus disease 2019 (COVID-19) immunization. It is reported by approximately half of the vaccine recipients, both in clinical trials and real-world data [1, 2]. Headache typically presents within the first 72 h post-vaccination and may be associated with additional symptoms, such as fatigue, fever, myalgia, arthralgia, or diarrhea [1, 2].

Several cases of cerebral venous thrombosis (CVT) have been reported in non-replicant adenovirus vector-based COVID-19 vaccine recipients (Oxford-AstraZeneca ChAdOx1-S and Johnson & Johnson (J&J) Janssen Ad26.COV2S) [3–7]. The association was based on the presence of thrombocytopenia [3–8], anti-platelet factor 4 antibodies [6, 7], and multiple organ thrombosis (vaccine-induced immune thrombotic thrombocytopenia (VITT)) [3–8]. There was a higher mortality rate [3–8] than in other CVT series reported in the literature and an increased standardized morbidity ratio (SMR) in patients aged between 30 and 49 [3]. SMR is also known as observed-to-expected analysis, which analyses the ratio between the observed number of cases in the population over the number of cases that would be expected based on the baseline incidence according to databases and prior studies [9, 10].

Headache is the most frequent symptom in CVT, and it may occur isolated or accompanied by other symptoms [9–11]. As in other secondary headache disorders, CVT may be recognized by the presence of red flags [11, 12]. Herein we highlight a potential novel red flag based on the available scientific data that may help clinicians to properly identify cases of CVT following non-replicant adenovirus vector-based COVID-19 vaccines.

## Methods

This is an observational study with case-control design. The study population were vaccinated with non-replicant adenovirus vector-based vaccines. The case definition was CVT following COVID-19 non-replicant adenovirus vector-based vaccines. We screened PubMed for all published cases and case series between March 01 and April 30, 2021, and assessed specific reports from the United States Centers for Disease Control and Prevention and the European Medicines Agency providing patient-level data. The search term is available in the supplementary appendix. We assessed and extracted the following variables, which are presented as descriptive statistics: age, sex, use of contraceptives or hormone-replacement therapy, presence of headache, presence of additional symptoms, the interval between the vaccination and the first symptom, intracranial hemorrhage, and death. We compared the time elapsed between vaccination and the first clinical onset in patients with and without headache. As control group, we reviewed the United States Vaccine Adverse Event Reporting System (VAERS) [2] reports under the symptom “headache” following COVID-19 vaccination up to April 30, 2021, assessing the number of days between the immunization and the headache onset. In the statistical analysis, we assessed whether the presence of headache in patients with CVT was associated with higher probability of death or with higher probability of intracranial hemorrhage by univariate logistic regression, presented as odds ratio (OR) and 95% confidence interval (CI). The statistical signification threshold was 0.05 and statistical tests were two-tailed. No statistical power calculation was conducted prior to the study. Statistical analysis was done with SPSS version 26.0.

## Results

We identified 77 cases of CVT included in eight publications (supplementary appendix). Patients’ age was available in 71 cases, and was below 40 in 46.5%, below 60 in 95.8% and between 60 and 69 years in 4.2% cases. The majority of cases 61/68 (89.7%) were women. Five (5.2%) patients used contraceptives and one (1.3%) received estrogen therapy.

**Fig. 1:**
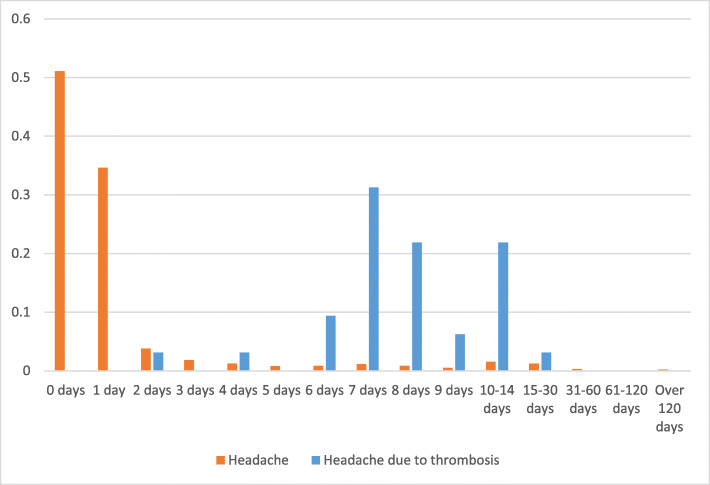
Temporal distribution of vaccine-related headache in the general population and vaccine-related cerebral venous sinus thrombosis headache. Orange bars represent the proportion of patients that present headache during each day after vaccination according to VAERS data^*2*^. Blue bars represent the proportion of patients with headache attributed to vaccine-related cerebral venous sinus thrombosis

The median time between the vaccination and the first symptom was 8 days (inter-quartile range (IQR) 7–12, range 1–19, *n* = 70). The presence of headache was described in 38/77 cases (49.4%). In 35/38 patients (92.1%), it was associated with other systemic or neurological symptoms. The CVT-related clinical symptoms started earlier in patients with headache (median 8, IQR 7–9.7, range 2–15) than in patients without headache (median 10, IQR 7–12.2, range 1–19), (*P* = 0.037, Mann-Whitney test). Figure 1 shows the days elapsed between vaccination and headache onset. Intracranial hemorrhage was present in 33/77 (42.9%) cases, multiple location thrombosis was reported in 19/77 (24.7%) cases, and 19/77 (24.7%) died. In patients with CVT, the presence of headache was associated with the presence of intracranial hemorrhage (odds ratio (OR) 7.45; 95% confidence interval (CI): 2.67–20.80, *P* < 0.001), but not with a higher odd of death (OR: 0.51, 95% CI: 0.18–1.47, *P* = 0.213).

## Discussion

Although headache is a common symptom after vaccination, it typically presents and resolves within the same day or a few days later [1, 2]. CVT, and other thrombotic complications in adenovirus-based vaccine recipients share a unique feature, the delayed presentation. In most of the reported cases symptoms started 1 week after immunization. Headache was not described in detail in most cases, but in the few it was, it was reported as severe, progressive and treatment-resistant, all of them well-known red flags [ 12].

Diagnostic delay is common in CVT [9]. Prompt treatment likely improves the clinical outcome, and therefore, every physician must be aware of the potential risk of vaccine related thrombotic complications. Given this knowledge and the possibility of vaccine-related CVT, patients with new-onset headache, 1 week after immunization, should receive a thorough clinical evaluation and be closely monitored. Non-contrast.

computed tomography (CT) may detect indirect findings of CVT, including hyperdense vein or sinus (cord sign), venous infarcts, brain oedema and intracranial hemorrhages; however, the optimal imaging test in case of suspicion are contrast brain CT with CT venogram, magnetic resonance imaging (MRI) with contrast and/or venogram, showing a filling defect in a venous sinus (empty delta sign) [10, 13].

The pathophysiology of thrombosis with thrombocytopenia syndrome has been recently discussed [7, 14, 15]. The role of anti-platelet factor-4 antibodies seem causative, inducing platelet activation, aggregation, and thrombosis, leading to a severe platelet consumption and thrombocytopenia. According to the existing evidence, an interim guideline published by the World Health Organization (WHO) states that the treatment should include the treatment of the immune-mediated phenomenon and adequate anticoagulation according to the existing evidence [14]. For the first, intravenous immunoglobulins seem to be the preferred option, while as anticoagulants, non-heparin-based anticoagulants must be used, while heparin-based anticoagulants and platelet infusion should be avoided [14].

## Conclusion

To conclude, delayed onset of headache following an adenovirus vector-based COVID-19 vaccine is associated with CVT. Patients with new-onset headache, 1 week after vaccination with an adenovirus vector-based vaccine, should receive a thorough clinical evaluation and CVT must be considered in the diagnostic work-up.

## Supplementary Information

**Additional file 1.**

## Data Availability

Datasheets are fully available to other researchers upon request to the corresponding author.
