# Supplementary material for: Delayed headache after COVID-19 vaccination: a red flag for vaccine induced cerebral venous thrombosis
Source: J Headache Pain. 2021 Sep 17;22(1):108. doi: 10.1186/s10194-021-01324-5 (PMC8446734; doi:10.1186/s10194-021-01324-5)
Supplement: Supplementary file 1 — Additional file 1. [file 10194_2021_1324_MOESM1_ESM.docx]

**Supplementary appendix:**

**Search term:**

(("Heparin induced thrombocytopenia"[tiab] OR

((prothrombotic OR thrombosis[tiab] OR thrombotic[tiab] OR thromboembolism[tiab][) AND ("thrombocytopenia](https://search.bvsalud.org/global-literature-on-novel-coronavirus-2019-ncov/resource/en/covidwho-1184841)"[tiab] OR thrombocytopenic[tiab])) )

AND systematic[sb] )

**References of the included articles:**

1. European Medicines Agency. Signal assessment report on embolic and thrombotic events (SMQ) with COVID-19 Vaccine (ChAdOx1-S [recombinant]) – Vaxzevria (previously COVID-19 Vaccine AstraZeneca) (Other viral vaccines) Version 08 April 2021. Available on: https://www.ema.europa.eu/en/documents/prac-recommendation/signal-assessment-report-embolic-thrombotic-events-smq-covid-19-vaccine-chadox1-s-recombinant_en.pdf
2. Tiede A, Sachs UJ, Czwalinna A, Werwitzke S, Bikker R, Krauss JK, Donnerstag FG, Weißenborn K, Höglinger GU, Maasoumy B, Wedemeyer H, Ganser A. Prothrombotic immune thrombocytopenia after COVID-19 vaccine. *Blood*. 2021. doi: 10.1182/blood.2021011958.
3. Greinacher A, Thiele T, Warkentin TE, Weisser K, Kyrle PA, Eichinger S. Thrombotic Thrombocytopenia after ChAdOx1 nCov-19 Vaccination. *N Engl J Med*. 2021. doi: 10.1056/NEJMoa2104840.
4. Scully M, Singh D, Lown R, Poles A, Solomon T, Levi M, Goldblatt D, Kotoucek P, Thomas W, Lester W. Pathologic Antibodies to Platelet Factor 4 after ChAdOx1 nCoV-19 Vaccination. *N Engl J Med*. 2021. doi: 10.1056/NEJMoa2105385.
5. Schultz NH, Sørvoll IH, Michelsen AE, Munthe LA, Lund-Johansen F, Ahlen MT, Wiedmann M, Aamodt AH, Skattør TH, Tjønnfjord GE, Holme PA. Thrombosis and Thrombocytopenia after ChAdOx1 nCoV-19 Vaccination. *N Engl J Med*. 2021. doi: 10.1056/NEJMoa2104882.
6. See I, Su JR, Lale A, Woo EJ, Guh AY, Shimabukuro TT, Streiff MB, Rao AK, Wheeler AP, Beavers SF, Durbin AP, Edwards K, Miller E, Harrington TA, Mba-Jonas A, Nair N, Nguyen DT, Talaat KR, Urrutia VC, Walker SC, Creech CB, Clark TA, DeStefano F, Broder KR. US Case Reports of Cerebral Venous Sinus Thrombosis With Thrombocytopenia After Ad26.COV2.S Vaccination, March 2 to April 21, 2021. *JAMA*. 2021. doi: 10.1001/jama.2021.7517.
7. Wolf ME, Luz B, Niehaus L, Bhogal P, Bäzner H, Henkes H. Thrombocytopenia and Intracranial Venous Sinus Thrombosis after "COVID-19 Vaccine AstraZeneca" Exposure. *J Clin Med*. 2021;10(8):1599. doi: 10.3390/jcm10081599.
8. Food and Drug Administration. Fact sheet for healthcare providers administering vaccine emergency use authorization of the Janssen COVIE-19 vaccine to prevent coronavirus disease 2019. Available on: https://www.fda.gov/media/146304/download
